# Successful Treatment of Carbamazepine-Induced Toxic Epidermal Necrolysis With Clinical Gastrointestinal Involvement: A Case Report

**DOI:** 10.3389/fped.2022.834037

**Published:** 2022-04-13

**Authors:** Le Quynh Chi, Nguyen Thi Van Anh, Nguyen Ngoc Quynh Le, Nguyen Thi Thu Ha, Hoang Minh Tien, Tran Minh Dien

**Affiliations:** ^1^Department of Rheumatology, Allergy, and Immunology, Vietnam National Children's Hospital, Hanoi, Vietnam; ^2^Surgical Intensive Care Unit, Vietnam National Children's Hospital, Hanoi, Vietnam

**Keywords:** gastrointestinal manifestation, SJS/TEN, pediatrics, Vietnam, diarrhea

## Abstract

**Background:**

Stevens-Johnson syndrome/toxic epidermal necrolysis (SJS/TEN) is a rare and life-threatening disease of the skin and mucosal surfaces. Although gastrointestinal manifestations in adults are potential prognostic factors for disease severity, there are limited data on such cases and their standard management in the pediatric population.

**Case Presentation:**

We herein report the case of an 8-year-old girl with a 1-year history of epilepsy, who presented with bilateral conjunctivitis and progressively widespread bullous, and pruritic eruption based on erythematous skin after administration of carbamazepine. A diagnosis of carbamazepine-induced TEN was made, and the drug was immediately discontinued. The result of genetic screening showed that the patient was positive for the HLA-B^*^15:02 allele. Then, her condition got worse by developing gastrointestinal involvement, including hematemesis and severe watery bloody diarrhea. A combination of the intravenous immunoglobulin and the appropriate dose of systemic steroids have contributed to a favorable outcome in this case. Multidisciplinary care of mucocutaneous involvement, supplemental nutrition, and fluid replacement was also critically warranted. This report aims to contribute to the current literature on TEN-related gastrointestinal manifestations in pediatrics and highlights the need for further investigations in determining the optimal treatment in such cases.

**Conclusion:**

In conclusion, we reported the successful treatment of TEN-related gastrointestinal manifestations in a pediatric patient, which should be critically considered in patients with SJS/TEN. Since it may significantly contribute to the poor prognosis of the illness, further investigations in determining standard management in such cases are necessary.

## Introduction

Stevens-Johnson syndrome/toxic epidermal necrolysis (SJS/TEN) is a rare and life-threatening disease of the skin and mucosal membranes. Based on affected body surface area (BSA), SJS involves <10% of BSA, TEN involves more than 30% of BSA, and SJS/TEN overlap involves 10 to 30% of BSA (1). Ocular and mucocutaneous lesions are the central and most common disease manifestations that are well-documented in the literature (2). Moreover, SJS/TEN also results in significant injuries of various organ systems, involving respiratory, gastrointestinal/hepatic, oral, otorhinolaryngologic, gynecologic/genitourinary, and renal complications (2).

Notably, gastrointestinal (GI) complications are not unusual in SIS/TEN and are potentially prognostic for disease severity (3). Clinical manifestations are nonspecific, such as GI bleeding (hematemesis, melena, and hematochezia), diarrhea, abdominal pain, abdominal distension, perforation, and dysphagia (3, 4). While previous reports of severe GI involvement in the literature reinforce its poor prognosis, standard management of such cases remains controversial. Therefore, the finding of GI involvement should be acknowledged in patients with SJS/TEN, and a multidisciplinary approach is warranted to avoid worsening digestive tract lesions and fatal outcomes (3, 5). To the best of our knowledge, SJS/TEN-related GI lesions have been comprehensively described in adults, but data on the pediatric population are sparse. Hence, we herein report the successful treatment of a case of toxic epidermal necrolysis presenting with hematemesis and hematochezia as gastrointestinal complications. This report aims to enrich the current literature on SJS/TEN-related gastrointestinal manifestations in pediatrics and highlight the need for further investigations in determining the optimal treatment in such cases.

## Case Report

An 8-year-old girl had a 1-year history of epilepsy, for which she was treated with oral sodium valproate at 200 mg every 8 h. Since her epilepsy had become uncontrollable since the last month, her parent stopped sodium valproate and switched to carbamazepine at 200 mg two times daily for the past 3 weeks. Four days before admission, she developed a sore throat, high non-intermittent fever, and erythematous rashes scattered on her body. Her parent thought it was just some viral infection and then gave her a combination of amoxicillin and clavulanic acid. However, after three days of treatment, the patient remained with a high fever and developed a widespread bullous, pruritic eruption from the trunk toward extremities on an erythematous base. Bilateral conjunctivitis, multiple erosions, and hemorrhagic crusts of the lips were noted. She was then transferred to Vietnam National Children's Hospital on the day fourth of illness.

**Figure 1:**
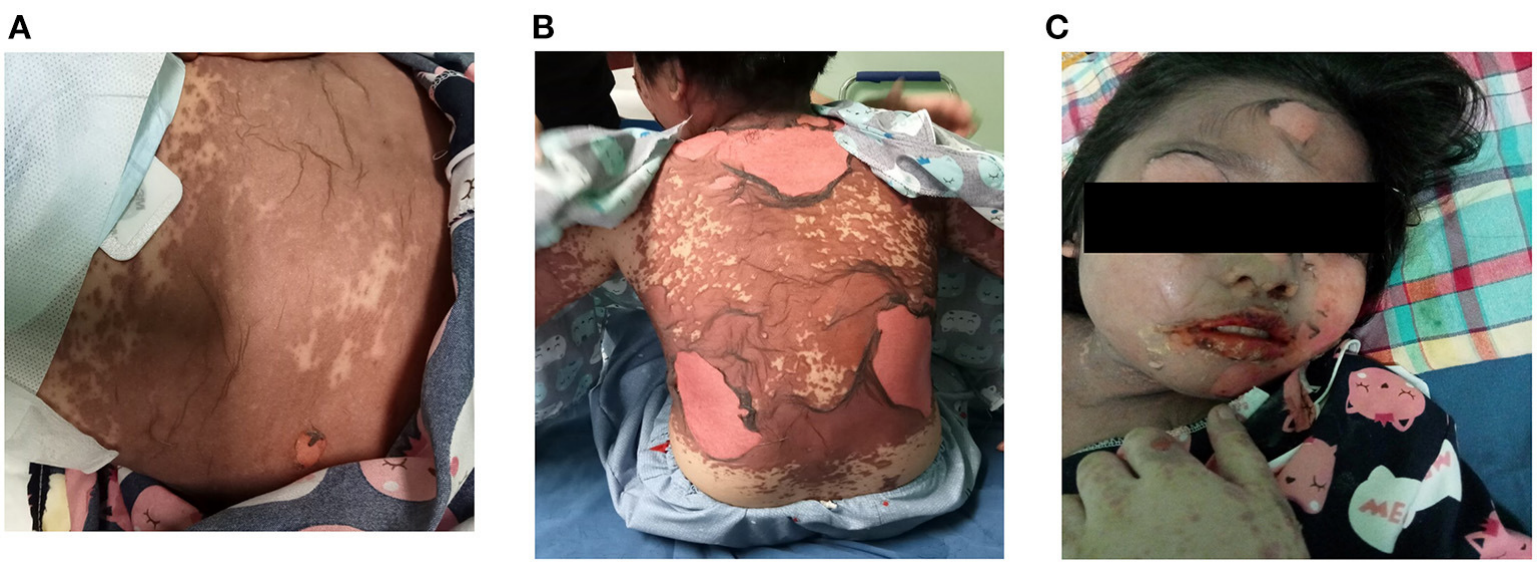
**(A,B)** A widespread epidermal detachment on the abdomen and back on hospital admission (day 4 of the illness). **(C)** Hemorrhagic crusting of the lips and skin desquamation.

On initial examination at admission, the 38-kg child had a widespread epidermal detachment on her body (Figure 1). Nikolsky's sign was positive. Remarkably, multiple oral ulcers of the buccal mucosa, tongue, soft palate, floor of the mouth, genitalia, and hemorrhagic crusting of the lips were also noted. Ophthalmic examination revealed conjunctivitis and sloughing of central corneal epithelium with positive fluorescein staining. Vital signs showed a temperature of 39°C, oxygen saturation of 100% at room air, tachycardia of 115 beats per minute (BPM), capillary refill time of <2 s, and noninvasive blood pressure (NIBP) of 125/75 mmHg. With normoactive bowel sounds, her abdomen was soft and not distended. Other organs were stable on examination. Her parent denied any previous history of drug allergy.

An initial total blood count revealed white blood cell (WBC) count of 8,100 cells/μl, red blood cell (RBC) count 508 × 103 cells/μl, platelet (PLT) count 358 × 103 cells/μl, aspartate aminotransferase (AST) 78 U/L, alanine aminotransferase (ALT) 43 U/L, urea (Ure) 14.9 mmol/L, creatinine (Cre, 138 μmol/L, blood urea nitrogen (BUN) to Cre ratio 26. 75, C-reactive protein (CRP) 180 mg/dl, albuminemia (ALB) 36 g/L, glycemia 6.2 mmol/L, serum bicarbonate 24.6 (mmol/L), fibrinogen 4.8 g/,; prothrombin time (PT) 14.3 s, activated partial thromboplastin time (APTT) 36.9 s, natremia, 131 mmol/L, and kalemia 3,4 mmol/L. Serum antibody titers failed to detect mycoplasma pneumonia, hepatitis virus (A, E, and C), HIV, and Aspergillus fungi. The result of the rapid hepatitis B test was negative. Microbiologic studies using polymerase chain reaction techniques revealed negative results of mycoplasma pneumonia, Epstein-Barr virus, cytomegalovirus, and Human herpesvirus-6 from various specimens. Blood and nasopharyngeal cultures were also negative.

According to typical manifestations and affected BSA, the patient was diagnosed with carbamazepine-induced TEN without skin biopsy and histopathology of the affected area. The algorithm of drug causality for epidermal necrolysis (ALDEN) was 6 points, which suggested a “very probable” causality of reaction (6). The genetic screening test showed that the patient was positive for the HLA-B^*^15:02 allele. The initial management comprised immediately withdrawing carbamazepine and administering crystalloid fluid replacement. With systemic steroids at 2 mg/kg/day, the intravenous immunoglobulin (IVIG) was administered at 1 g/kg/day for two consecutive days. The patient received broad-spectrum antibiotics with meropenem and linezolid for the suspected infection, which we could not entirely rule out. The ocular involvement was managed with topical antibiotics of tobramycin and artificial tears. Supportive care for mucocutaneous, genitourinary lesions, antacids, and supplemental nutrition was also warranted.

On the third day of hospitalization (7 days after cutaneous presentation), she developed hematemesis and diarrhea of 15–20 watery bloody stools per day. She remained with a high fever and progressed to extensive sloughing of cutaneous lesions. Physical examination showed a body temperature of 38.8°C, tachycardia of 128 BPM, NIBP of 115/72 mmHg, and pulse oximetry of 97% at room air. Soft, non-distended abdomen and no abdominal pain were recorded. Arterial blood gas analysis showed pH of 7.42, PaO2 of 85 mmHg, PaCO2 of 33 mmHg, and bicarbonate of 22.6. Conventional coagulation tests were as follows: PLT count 223 × 103 cells/μl, PT 14 s, APTT 36 s, fibrinogen 4.7 g/L, and D-dimer 1,210 ng/ml FEU. Other remarkable results of laboratory tests were hyponatremia of 128 mmol/L, hypokalemia of 3.2 mmol/L, hypoalbuminemia of 21.9 g/L, a low hemoglobin level of 96 g/L, and procalcitonin of 1.68 ng/ml. A diagnostic workup of stool samples showed a strong positive presence of white blood cells and erythrocytes. However, a stool real-time PCR failed to detect 24 infectious pathogens, involving Vibrio vulnificus, Vibrio parahaemolyticus, Vibrio cholera, Campylobacter spp. (*C. jejuni, C. upsaliensis*, and *C. coli)*, Salmonella spp., Clostridium difficile (tcdA/tcdB), Yersinia enterocolitica, EAEC, ETEC, STEC, STEC O157:H7, EPEC, EIEC, Plesiomonasshigelloides, Norovirus GI and GII, Rotavirus A, Astrovirus, Spovirus GI, GII, GIV, GV, Entamoebahistolytica, Cryptosporidium spp., Giardia lamblia, and Cyclosporacayetanensis. Stool cultures were also negative. The Score for Toxic Epidermal Necrolysis was 3 points (tachycardia > 120, serum urea > 10 mmol/L, and initial detachment > 10%), giving a probability of in-hospital mortality of 35.3% (7). Her tachycardia responded well to 20 ml/kg of crystalloid fluid replacement over 30 min and 2.5 L of daily fluid compensation. Considering the high risk of hemorrhage and perforation, we decided to delay digestive tract endoscopy and biopsy procedures. We maintained 2 mg/kg/day of methylprednisolone for another 6 days. Also, additional intravenous IVIG at 1 g/kg/day was indicated for two consecutive days. The patient required electrolyte adjustment and a TPN supplement for daily nutrition.

On the ninth day after admission, her diarrhea declined to <10 watery stools per day without hematochezia. Because of non-progressive mucocutaneous and ocular lesions, the methylprednisolone dose was gradually tapered off on the tenth day of hospitalization. The patient responded well to the partial enteral nutrition provided on day 12. After 18 days of admission, she was discharged home to continue the tapering-off of oral steroid therapy and was required to return to the outpatient clinic for long-term follow-up.

## Discussion

The SJS/TEN is a life-threatening immunologically-mediated reaction with high mortality risk, although it has a low global incidence (1–2 cases per million population per year) (8–10). The sparse literature of the pediatric population showed lower incidence than in adults, but SJS/TEN continues to be one of the significant burdens in pediatrics worldwide, particularly in developing countries with a poor infrastructure setting (10, 11). The SJS/TEN is often triggered by drugs (antiepileptic drugs, antibiotics, chemotherapy, and non-steroidal anti-inflammatory drugs - NSAIDs) or infectious pathogens (viral and bacterial organisms), but no trigger is identified in 10% of cases (12). In our cases, carbamazepine had been started three weeks prior to symptoms' onset time, and microbiologic investigations failed to detect evidence of infections. The patient was also positive for the HLA-B^*^15:02 allele in the genetic screening test. Along with manifestations in the conjunctiva, mucosa, trachea, and genitourinary tract, GI tract involvement is one of the extracutaneous complications of SJS/TEN (3). While severe SJS/TEN-associated GI involvement and its poor prognosis in adult patients have been reported, surveillance data for estimating the contribution and management of GI complications in pediatric SJS/TEN remain limited. Thus, more robust data on GI manifestations of childhood SJS/TEN are needed to enrich the current literature and standardize pediatric-specific therapeutic strategies in such cases. Our study is the first report from a developing country in Southeast Asia presenting the successful treatment of TEN-related GI involvement in the pediatric literature to the best of our knowledge.

Previous data on adults have reported that GI complications are not uncommon in SJS/TEN, and are only noted in about 10% of patients with SJS/TEN. Severe GI complications occur rarely but can potentially mark a poor prognosis, with 44% of mortality (3, 12). Clinical manifestations are nonspecific and often have an onset of within 1–3 weeks following rash/mucosal lesions (3, 12–14). In a study by Jha et al. (3), these include GI bleeding (hematemesis, melena, and hematochezia) (68%), diarrhea (52%), abdominal pain (40%), abdominal distension (12%), perforation (12%), and vomiting (8%). Silent or asymptomatic GI lesions have not been reported, probably because a routine workup of endoscopy in SJS/TEN has not been performed. In our case, the patient also presented common symptoms of hematemesis and massively watery bloody diarrhea on the seventh day after the onset of illness. We recommended that stool samples should be investigated to exclude infectious pathogens in such a scenario.

Evaluation of the extent of GI involvements and histopathological characteristics in SJS/TEN might be obtained by GI tract endoscopy and with biopsy procedures. Hebra and Kaposi first in 1874 (15) and Osler later in 1895 (16) described gastrointestinal involvements with visceral manifestations of erythema multiforme, similar to skin lesions. Other endoscopic findings include hyperemia, congestion, irregular friable mucosa, adherent fibrin-like exudate erosions, superficial or deep ulcerations, and necrotic plaque formation with mucosal sloughing (17). Histologically, the early stage suggests epithelial necrosis without any abscess and lymphomononuclear cell infiltrations, which progress to severe necrotic ulcerations, lymphomononuclear infiltration of the lamina propria, and inflamed granulation tissue in the later stage (17, 18). Previous studies have reported that esophagitis, gastritis, duodenitis, jejunitis, ileitis, and colitis are common GI lesions (60%), and that the large bowel is the most common site of ulcers followed by the small bowel and stomach (3). Other reports regarded GI as more frequently occurring in the mouth and esophagus than in the small intestine and colon (12). Alongside hematemesis and extensively watery-bloody diarrhea, hypoalbuminemia was also seen in our case. According to previous reports, diarrhea, protein-losing enteropathy, and hypoalbuminemia might reveal multiple ileal strictures, pseudodiverticular sacs, and pseudomembranes formation (19, 20). Therefore, because of strictly considering the high risk of gastrointestinal hemorrhage and perforation and currently suspected infection (CRP of 180 mg/dl and PCT of 1.68 ng/ml), GI endoscopy and biopsy were not performed, and that is a limitation in our report. Deciding GI endoscopy and biopsy for patients with TEN is a challenge since patients with TEN, particularly pediatrics with GI manifestations, may include hemodynamic instability and often require aggressive fluid and electrolyte management. Moreover, GI endoscopy and biopsy should be performed cautiously to prevent pathogen invasions by disrupting the damaged intestinal mucosal barrier.

The pathogenesis for SJS/TEN-related GI involvement has been unknown, but probably similar to T–cell-mediated hypersensitivity reactions responsible for skin lesions (17). Although some situations require surgical interventions, supportive and disciplinary care is the cornerstone of management of SJS/TEN-associated GI manifestations. However, optimal therapeutic options, such as systemic corticosteroids, intravenous immunoglobulin, plasmapheresis, plasma exchange, cyclosporine, and TNF-α antagonists, are still controversial (21). While some data support systemic steroids as standard care for treatment of SJS/TEN (3, 21, 22), clinicians have raised concerns about mucosal sloughing exacerbation, GI bleeding, GI perforation, and risk of infection (21). Regarding the rationale of IVIG transfusion, high doses of IVIG could block the apoptosis resulting from the interaction between the Fas–Fas ligand and epidermal cells in SJS/TEN (23). To the best of our knowledge, only a systematic review and meta-analysis of literature reported that the efficacy of IVIG in lowering the mortality rate of pediatrics with SJS/TEN might be significantly higher than that of adults, with a mortality rate of 0 and 21.6%, respectively (*P* = 0.001) (24). Recently, as a central mediator in the pathogenesis of inflammatory bowel disease, tumor necrosis factor-alpha (TNF-α) inhibitors might be possibly therapeutic in GI-related SJS/TEN (13, 21). With its efficacy of rapid re-epithelialization, cyclosporin A could reduce the mortality rate in adults and children with SJS/TEN (25). However, the results of previous studies have remained controversial; further well-designed investigations should be undertaken to determine the optimal management of SJS/TEN and its GI involvement. For our case, the patient received a 6-day course of 2 mg/kg/day of steroids and a total of 1 g/kg/day for 4 consecutive days in the acute phase. Given the suspected infection, strict infection control with broad-spectrum antibiotics and appropriate tapering-off of doses of the steroids were warranted. Determinants of successful management of “such potentially fatal burned patient” also included early aggressive fluid replacement, electrolyte balance, topical remedy, peptic ulcer prophylaxis, and early total parenteral nutrition (TPN) supplement.

## Conclusion

In conclusion, we reported the successful treatment of TEN-related gastrointestinal manifestations in pediatrics, which should be critically considered in patients with SJS/TEN. This highlights the need for further investigations to determine the optimal treatment for such cases, since it may significantly contribute to the poor prognosis of the illness.

## Data Availability Statement

The original contributions presented in the study are included in the article/supplementary files, further inquiries can be directed to the corresponding author.

## Ethics Statement

The studies involving human participants were reviewed and approved by Institutional Review Board of Vietnam National Children's Hospital. Written informed consent to participate in this study was provided by the participants' legal guardian/next of kin. Written informed consent was obtained from the individual(s), and minor(s)' legal guardian/next of kin, for the publication of any potentially identifiable images or data included in this article.

## Author Contributions

LC, NA, NL, and TD contributed to the conception and design of the study. NH wrote the first draft of the manuscript. HT and NH collected the data and wrote sections of the manuscript. All authors contributed to manuscript revision and read and approved the submitted version.

## Conflict of Interest

The authors declare that the research was conducted in the absence of any commercial or financial relationships that could be construed as a potential conflict of interest.

## Publisher's Note

All claims expressed in this article are solely those of the authors and do not necessarily represent those of their affiliated organizations, or those of the publisher, the editors and the reviewers. Any product that may be evaluated in this article, or claim that may be made by its manufacturer, is not guaranteed or endorsed by the publisher.
